# Safe2Play in youth ice hockey: injury profile and risk factors in a 5-year Canadian longitudinal cohort study

**DOI:** 10.1080/07853890.2024.2385024

**Published:** 2024-08-27

**Authors:** Paul H. Eliason, Jean-Michel Galarneau, Shelina Babul, Martin Mrazik, Stephan Bonfield, Kathryn J. Schneider, Brent E. Hagel, Carolyn A. Emery

**Affiliations:** a Faculty of Kinesiology, Sport Injury Prevention Research Centre, University of Calgary, Calgary, Canada; b Alberta Children’s Hospital Research Institute, University of Calgary, Calgary, Canada; c O’Brien Institute for Public Health, University of Calgary, Calgary, Canada; d Hotchkiss Brain Institute, University of Calgary, Calgary, Canada; e Faculty of Medicine, British Columbia Injury Research and Prevention Unit, University of British Columbia, Vancouver, Canada; f Faculty of Education, University of Alberta, Edmonton, Canada; g Department of Clinical Neurosciences and Division of Physical Medicine and Rehabilitation, Cumming School of Medicine, University of Calgary, Calgary, Canada; h Community Health Sciences, Cumming School of Medicine, University of Calgary, Calgary, Canada; i Pediatrics, Cumming School of Medicine, University of Calgary, Calgary, Canada

**Keywords:** Ice hockey, risk factors, injury, adolescent, injury prevention

## Abstract

**Introduction:**

Ice hockey is a popular global sport with growing participation for boys and girls yet remains a high-risk sport for injury. While the evidence for some risk factors, such as bodychecking policy is well established, other risk factors such as player sex have been understudied. The objectives of this study were to examine factors associated with rates of game-related injury, game-related injury resulting in >7 days of time-loss, and practice-related injury in youth ice hockey.

**Materials and methods:**

Safe2Play was a five-year prospective cohort study (2013–2018). All injuries were identified using validated injury surveillance methodology. Multilevel Poisson regression (adjusting for cluster by team and including multiple imputation of missing covariates) was used to estimate incidence rate ratios (IRRs) for player sex, age group, bodychecking policy, year of play, level of play, weight, previous injury within last 12 months, previous lifetime concussion history, and position.

**Results:**

A total of 4418 male and female ice hockey players (representing 6584 player-seasons) participating in under-13 (ages 11–12), under-15 (ages 13–14) and under-18 (ages 15–17) age groups were recruited. There were 1184 game-related and 182 practice-related injuries. Factors associated with game-related injury included female sex (IRR = 1.57; 95% CI: 1.18–2.08), previous injury (IRR = 1.46; 95% CI: 1.26–1.70) and lifetime concussion history (IRR = 1.41; 95% CI: 1.23–1.62). Goaltenders had a lower rate of injury (IRR = 0.54; 95% CI: 0.40–0.72) relative to forwards, as did players exposed to policy disallowing bodychecking in games (IRR = 0.44; 95% CI: 0.35–0.55). Female sex (IRR = 1.90; 95% CI: 1.10–3.28) and lifetime concussion history were also significantly associated with practice-related injury (IRR = 1.53; 95% CI: 1.08–2.18).

**Conclusions:**

Based on data from a large 5-year Canadian youth ice hockey longitudinal cohort, several factors associated with injury were identified. Future injury prevention strategies should consider age, sex, previous concussion and injury history, and body checking leagues. Future research in female youth ice hockey including female-only leagues should be a priority to inform prevention strategies in this understudied population.

## Introduction

Ice hockey is a popular winter sport and has grown in participation globally, particularly for females, over the past decade [1,2]. There are many known benefits to participating in sport such as improved physical, social and mental health benefits [3]. Unfortunately, participation in ice hockey is associated with a high burden of injury [4–6]. In Canada, ice hockey accounts for over 10% of all youth sport-related injuries [5]. Concussion incidence rates in ice hockey are among the highest in youth sports [4], accounting for between 18 and 66% of all ice hockey-related injuries [7–10].

Previous youth ice hockey studies have identified risk factors for injury such as policy permitting bodychecking [9–16]. This research has informed policy disallowing bodychecking in games in the under-13 age group (ages 11–12) nationally in the USA and Canada (USA Hockey 2011; Hockey Canada 2013), as well as in non-elite levels of play in older age categories (ages 13–17) [7,10,13,14]. These policy changes have prevented thousands of injuries to date and subsequently contributed to considerable health care cost savings [7,10,13,14,17–19]. While the evidence is strong for bodychecking policy and some other risk factors, like previous injury and concussion history, the evidence for other factors such as sex, weight, level of play, and player position is inconsistent or understudied. This may be due in part to differing injury definitions, methodological discrepancies and inconsistent reporting [12]. Further, the literature to date has primarily focused on game-related injury with little attention to practice-related injury.

Safe2Play was a large 5-year prospective cohort study in Canadian youth ice hockey players and provides an ideal opportunity to examine factors associated with injury for games and practices. As such, the purpose of this study was to examine the association between several potential risk factors for game-related injury, game-related injury resulting in >7 days of time-loss, and practice-related injury rates in youth ice hockey players aged 11–17.

## Materials and methods

### Design

Safe2Play was a 5-year prospective cohort study conducted from 2013/14 to 2017/18 in two western Canada provinces (Alberta and British Columbia). The cohort included male and female players from all levels of play in under-13 (ages 11–12), under-15 (ages 13–14) and under-18 (ages 15–17) age groups. This included players in leagues either allowing or disallowing bodychecking. Eligible females played in either ‘female-only’ leagues, where policy does not permit bodychecking at any age group or level of play, or on primarily male-participant teams where bodychecking policy was league dependent. The cohort utilised an inclusive sampling approach where all regional hockey associations were invited to participate. Individual teams were then contacted from those associations that agreed to participate. Teams were included if they agreed to participate and could identify a person willing to report weekly player participation and any injuries in games and practices. All players from eligible teams could participate if they provided written informed consent (player or parent) and had no previous injury or illness that prevented full participation at the beginning of the playing season. The recruitment process details have been previously published [10,13,14]. As this was a secondary data analysis, there was no *a priori* sample size calculation. Sample size details from the original study have been previously published [8]. Ethical approval was received from research ethics boards at the University of Calgary (Ethics ID: REB14-0348 and REB14-2209), University of Alberta (Ethics ID: REB Pro00024093) and the University of British Columbia (Ethics ID: CW14-0304/H14-01894).

### Procedures

The previously validated youth ice hockey injury surveillance methodology included a preseason baseline questionnaire, recording of weekly playing exposure in games and practices, and injury details [20]. Each team designate (typically the team manager) collected weekly playing exposure and identified players with injuries. Injuries must have resulted in medical attention, the inability to complete a session, or time-loss from playing to be included. A study athletic therapist and/or physiotherapist confirmed the information on all injuries that were reported by the team designate. Further details of the validated injury surveillance system have been previously published [9,10,13,17]. Players with a suspected concussion were referred to a study sport medicine physician within 72 h for diagnosis and management. Concussion definition and a standardised follow-up and return to play protocol based on the 4th (2013–2016) and 5th (2017–2018) International Conferences on Concussion in Sport (Zurich 2013 and Berlin 2016 Consensus Statements) was followed by all study physicians [21,22]. To ensure consistency across diagnosis and return to sport processes as per the latest International Statement on Concussion in Sport [21,22], meetings with study physicians were held at the start of each season. Study therapists validating the injury report forms also completed standardised training at the start of each season.

The outcomes of interest for this study were the incidence of game-related injury, game-related injury that resulted in >7 days of time-loss, and all practice-related injuries. The seven-day cut-point for injury has been supported in the literature to differentiate between more severe injury and allows for comparison with previous youth ice hockey studies [7,9,10,13,15,16,20,23,24]. Since most concussions typically resolve within 4 weeks in youth [21,22], a sensitivity analysis was also conducted restricting injury that resulted in >7 days of time loss to musculoskeletal injury only (i.e. excluding concussion).

### Statistical analyses

The baseline characteristics of the cohort were described by age group and injury status. When weekly playing exposure was missing, an estimation approach based on weekly means was performed within participant, team, or city and division of play. This technique is a recommended method for dealing with missing playing exposure in youth ice hockey and is supported by several previous studies [7,10,13,25]. Crude rates of game-related injury, game-related injury resulting in >7 days of time-loss, and all practice-related injuries (offset for game-hours for game-related injury and practice-hours for practice-related injury) were estimated for each age group with 95% Poisson confidence intervals adjusted for cluster by team. For each age group, crude game and practice-related injury rates were calculated by injury location and type with either exact Poisson 95% CIs (when injury numbers <10) or adjusted for clustering by team (numbers ≥10).

Separate multiple multilevel Poisson regression models were used to evaluate potential risk factors associated with each injury outcome. Each model was adjusted for the following important covariates as suggested by previous youth ice hockey studies [7,8,10,12–16]: age group (under-13, under-15, under-18), bodychecking policy (permitted, not permitted), year of play (1st, 2nd or 3rd), level of play (elite divisions of play (top 20–30% by division of play depending on age group), sub-elite (lower 70–80%)), sex (male, female), player weight (kg), previous injury in the last 12 months (yes, no), previous lifetime concussion history (yes, no) and player position (forward, defence, goalie). Non-linearity of weight was explored by itself and with an interaction term within each age group (*α* = 0.05). Player exposure hours were used as an offset in each model (game-hours for game-related outcomes and practice-hours for the practice outcome), and a random effect at the team level was examined to account for clustering. Missing covariate data were imputed using multiple imputation using chained equations with 30 imputations completed. All analyses were completed using Stata MP (release 17; with copyright license) [26].

## Results

The Safe2Play cohort recruited a total of 4418 players. Of those, 1344 participated in more than one season of play and resulted in a combined 6584 player-seasons. The median number of players recruited per team was 14 (range: 2–20). There were also an additional 1027 individual players (representing 1308 player-seasons) that were the only members of a team to participate. The median number of weeks players were followed was 20 (range: 2–31) and varied depending on when they were recruited during the playing season. Most players (84.6%; 5565/6581) had at least one week of game exposure estimated. The median number of game weeks imputed per player was 3 (first quartile: 1, third quartile: 14).

Table 1 summarises the baseline characteristics of the player-seasons by age group and injury status. Of interest, ‘female-only’ leagues had a higher proportion of injured players (119/431; 27.6%) than females playing on predominantly male-based teams (22/168; 13.1%).

**Table 1.** Baseline characteristics for under-13, under-15 and under-18 youth ice hockey players over five seasons (2013–2018).

	Under-13		Under-15		Under-18	
	Injured (*n*^c^ = 159)	No injury (*n*^c^ = 1410)	Injured (*n*^c^ = 591)	No injury (*n*^c^ = 2486)	Injured (*n*^c^ = 439)	No injury (*n*^c^ = 1499)
City, *n* (%)						
Calgary	150 (94.34)	1292 (91.63)	392 (66.33)	1549 (62.31)	276 (62.87)	832 (55.50)
Calgary surrounding area	1 (0.63)	29 (2.06)	13 (2.20)	57 (2.29)	24 (5.47)	45 (3.00)
Edmonton	8 (5.03)	88 (6.24)	142 (24.03)	520 (20.92)	105 (23.92)	291 (19.41)
Vancouver	0 (0)	0 (0)	42 (7.11)	321 (12.91)	34 (7.74)	331 (22.08)
Okanagan region	0 (0)	1 (0.07)	2 (0.34)	39 (1.57)	0 (0)	0 (0)
Year, *n* (%)						
2013–2014	80 (50.31)	652 (46.24)	19 (3.21)	71 (2.86)	0 (0)	0 (0)
2014–2015	48 (30.19)	340 (24.11)	180 (30.46)	830 (33.39)	0 (0)	0 (0)
2015–2016	11 (6.92)	83 (5.89)	198 (33.50)	702 (28.24)	199 (45.33)	709 (47.30)
2016–2017	13 (8.18)	172 (12.20)	101 (17.09)	490 (19.71)	133 (30.30)	410 (27.35)
2017–2018	7 (4.40)	163 (11.56)	93 (15.74)	393 (15.81)	107 (24.37)	380 (25.35)
Sex, *n* (%)						
Male	141 (88.68)	1295 (91.84)	519 (87.82)	2252 (90.59)	388 (88.38)	1390 (92.73)
Female	18 (11.32)	115 (8.16)	72 (12.18)	234 (9.41)	51 (11.62)	109 (7.27)
Sex within league, *n* (%)						
Males in mainly male leagues	141 (88.68)	1295 (91.84)	519 (87.82)	2252 (90.59)	388 (88.38)	1390 (92.73)
Females in ‘female-only’	10 (6.29)	38 (2.70)	62 (10.49)	183 (7.36)	47 (10.71)	91 (6.07)
Females in mainly male-based leagues	8 (5.03)	77 (5.46)	10 (1.69)	51 (2.05)	4 (0.91)	18 (1.20)
Anthropometrics						
Height, cm, median (Q1, Q3)	152.4 (147.0, 158.3)	152.2 (147.0, 157.5)	165.1 (157.6, 172.0)	165.3 (159.0, 172.5)	177.8 (171.2, 180.3)	177.0 (171.40, 182.9)
Missing, *n* (%)	20 (12.58)	74 (5.25)	187 (31.64)	875 (35.20)	116 (26.42)	512 (34.16)
Weight, kg, median (Q1, Q3)	42.4 (35.6, 48.8)	41.4 (36.5, 47.0)	54.4 (46.5, 62.5)	54.0 (46.7, 61.2)	68.0 (61.2, 74.8)	68.0 (61.2, 76.0)
Missing, *n* (%)	21 (13.21)	55 (3.90)	192 (32.49)	838 (33.71)	118 (26.88)	499 (33.29)
Level of play, *n* (%)						
Elite (top 20–30%)	52 (32.70)	470 (33.33)	193 (32.66)	551 (22.16)	165 (37.59)	425 (28.35)
Sub-elite (lower 70–80%)	95 (59.75)	848 (60.14)	376 (63.62)	1806 (72.65)	262 (59.68)	1022 (68.18)
Missing	12 (7.55)	92 (6.52)	22 (3.72)	129 (5.19)	12 (2.73)	52 (3.47)
Bodychecking policy, *n* (%)						
Allowed	NA	NA	443 (74.96)	1512 (60.82)	332 (75.63)	897 (59.84)
Prohibited	159 (100)	1410 (100)	132 (22.34)	853 (34.31)	99 (22.55)	561 (37.42)
Missing	0 (0)	0 (0)	16 (2.71)	121 (4.87)	8 (1.82)	41 (2.74)
Year of play, *n* (%)						
First	69 (43.40)	649 (46.03)	277 (46.87)	1196 (48.11)	172 (39.18)	618 (41.23)
Second	90 (56.60)	755 (53.55)	289 (48.90)	1176 (47.30)	128 (29.16)	520 (34.69)
Third	NA	NA	NA	NA	69 (15.72)	208 (13.88)
Missing	0 (0)	6 (0.43)	25 (4.23)	114 (4.59)	70 (15.95)	153 (10.21)
Position, *n* (%)						
Forward	82 (51.57)	740 (52.48)	319 (53.98)	1242 (49.96)	226 (51.48)	713 (47.57)
Defence	57 (35.85)	441 (31.28)	186 (31.47)	726 (29.20)	128 (29.16)	407 (27.15)
Goalie	9 (5.66)	133 (9.43)	29 (4.91)	216 (8.69)	27 (6.15)	153 (10.21)
Missing	11 (6.92)	96 (6.81)	57 (9.64)	302 (12.15)	58 (13.21)	226 (15.08)
Previous injury^a^						
No	69 (43.40)	884 (62.70)	229 (38.75)	1236 (49.72)	141 (32.12)	707 (47.16)
Yes	60 (37.74)	329 (23.33)	238 (40.27)	752 (30.25)	219 (49.89)	547 (36.49)
Missing	30 (18.87)	197 (13.97)	124 (20.98)	498 (20.03)	79 (18.00)	245 (16.34)
Previous concussion^b^						
No	91 (57.23)	975 (69.15)	287 (48.56)	1509 (60.70)	154 (35.08)	735 (49.03)
Yes	65 (40.88)	415 (29.43)	270 (45.69)	875 (35.20)	255 (58.09)	678 (45.23)
Missing	3 (1.89)	20 (1.42)	34 (5.75)	102 (4.10)	30 (6.83)	86 (5.74)

Q1: first quartile; Q3: third quartile.

^a^ Previous injury or concussion 12 months prior to baseline test.

^b^ Previous concussion lifetime.

^c^ Sum of *n* is 6584, given that it is player-seasons (1344 players participated in more than one season).

Table 2 summarises the injury outcomes for each age group. The proportion of game-related injuries that resulted in >7 days of time-loss was similar for those under-13 (79/138; 57.2%), under-15 (359/594; 60.4%) and under-18 (304/452; 67.3%). The rates of game-related injury and game-related injury resulting in >7 days of time-loss was highest in the under-18 age group followed by under-15 and lowest in under-13. The rates of practice-related injury were markedly lower compared with game-related rates but still highest in the under-18 age group.

**Table 2.** Summary of injury outcomes for under-13, under-15 and under-18 youth ice hockey players.

	Under-13	Under-15	Under-18
Number of game-related injuries	138	594	452
Number of game-related injuries with >7 days of time-loss	79	359	304
Player game participation hours	59891.21	128321.3	80505.3
Game-related injury rate, injuries per 1000 player game-hours (95% CI)^a^	2.30 (1.91, 2.78)	4.63 (4.12, 5.20)	5.61 (4.86, 6.48)
Game-related injury rate with >7 days of time-loss, injuries per 1000 player game-hours (95% CI)^a^	1.32 (1.07, 1.63)	2.80 (2.45, 3.20)	3.78 (3.25, 4.39)
Number of practice-related injuries	35	79	68
Player practice participation hours	53765.45	120814.4	75368.29
Practice-related injury rate, injuries per 1000 player practice-hours (95% CI)^a^	0.65 (0.47, 0.91)	0.65 (0.51, 0.84)	0.90 (0.68, 1.20)

^a^ Crude rates with 95% Poisson CIs adjusted for cluster by team (offset by game or practice hours).

The most common game-related injury location was the head/face for each age group (Table 3). The shoulder/clavicle was the next most common region of injury for players under-18 and under-15, and these rates were markedly higher compared with those under-13. Concussion was the most common game-related injury type for each age group. The rates of more severe injury types such as joint sprains/dislocations and fractures were also notably higher in under-18 and under-15 groups compared with those under-13. When practice-related injury details were known, the head/face was the most common region of injury for all age groups and concussion was the most common injury type (Table 4).

**Table 3.** Number and rates of game-related injuries per 1000 player game-hours among under-13, under-15 and under-18 youth ice hockey players by injury type and location.

	Under-13		Under-15		Under-18	
	Number (/138)	Rate^a^ (95% CI)	Number (/594)	Rate^a^ (95% CI)	Number (/452)	Rate^a^ (95% CI)
Injury location						
Head/face	88	1.47 (1.17, 1.85)	290	2.26 (1.96, 2.61)	185	2.30 (1.95, 2.71)
Neck/throat	3	0.05 (0.01, 0.15)	18	0.14 (0.09, 0.22)	8	0.10 (0.04, 0.20)
Shoulder/clavicle	4	0.07 (0.02, 0.17)	55	0.43 (0.32, 0.58)	64	0.80 (0.61, 1.04)
Upper arm/elbow/forearm	6	0.10 (0.04, 0.22)	20	0.16 (0.10, 0.25)	11	0.14 (0.07, 0.26)
Wrist/hand	4	0.07 (0.02, 0.17)	42	0.33 (0.23, 0.46)	38	0.47 (0.33, 0.67)
Back/side	4	0.07 (0.02, 0.17)	17	0.13 (0.08, 0.23)	17	0.21 (0.13, 0.35)
Chest/ribs/abdomen	3	0.05 (0.01, 0.15)	13	0.10 (0.06, 0.18)	6	0.07 (0.03, 0.19)
Pelvis/hips/groin/upper leg	4	0.07 (0.02, 0.17)	25	0.19 (0.12, 0.31)	26	0.32 (0.18, 0.57)
Knee	7	0.12 (0.05, 0.24)	32	0.25 (0.17, 0.36)	36	0.45 (0.30, 0.66)
Lower leg/ankle/foot	9	0.15 (0.07, 0.29)	25	0.19 (0.13, 0.29)	19	0.24 (0.14, 0.39)
Other	1	0.02 (0.00, 0.09)	4	0.03 (0.01, 0.08)	2	0.02 (0.00, 0.09)
Missing/unknown	5	0.08 (0.03, 0.19)	53	0.41 (0.29, 0.58)	40	0.50 (0.35, 0.70)
Injury type						
Contusion	19	0.32 (0.19, 0.53)	47	0.37 (0.27, 0.50)	33	0.41 (0.27, 0.63)
Concussion	86	1.44 (1.14, 1.81)	287	2.24 (1.94, 2.58)	181	2.25 (1.90, 2.66)
Joint/ligament sprain/dislocation	12	0.20 (0.11, 0.36)	60	0.47 (0.35, 0.62)	76	0.94 (0.71, 1.25)
Fracture	5	0.08 (0.03, 0.19)	75	0.58 (0.45, 0.76)	56	0.70 (0.50, 0.96)
Muscle strain/tendinitis	7	0.12 (0.05, 0.24)	53	0.41 (0.30, 0.57)	40	0.50 (0.36, 0.69)
Abrasion/bleeding/burn/cut/blister	1	0.02 (0.00, 0.09)	3	0.02 (0.00, 0.07)	5	0.06 (0.02, 0.14)
Other	0	0.00 (0.00, 0.06)	7	0.05 (0.02, 0.11)	6	0.07 (0.03, 0.16)
Missing/unknown	8	0.13 (0.06, 0.26)	62	0.48 (0.36, 0.65)	55	0.68 (0.51, 0.91)

^a^ Crude rates per 1000 game-hours with 95% CIs adjusted for cluster by team. Exact CIs were calculated when number was less than 10.

**Table 4.** Number and rates of practice-related injuries per 1000 player practice-hours among under-13, under-15 and under-18 youth ice hockey players by injury type and location.

	Under-13		Under-15		Under-18	
	Number (/35)	Rate^a^ (95% CI)	Number (/79)	Rate^a^ (95% CI)	Number (/68)	Rate^a^ (95% CI)
Injury location						
Head/face	20	0.37 (0.25, 0.56)	35	0.29 (0.21, 0.40)	10	0.13 (0.07, 0.26)
Neck/throat	1	0.02 (0.00, 0.10)	0	0.00 (0.00, 0.03)	0	0.00 (0.00, 0.05)
Shoulder/clavicle	0	0.00 (0.00, 0.07)	4	0.03 (0.01, 0.08)	9	0.12 (0.05, 0.23)
Upper arm/elbow/forearm	0	0.00 (0.00, 0.07)	1	0.01 (0.00, 0.05)	2	0.03 (0.00, 0.10)
Wrist/hand	2	0.04 (0.00, 0.13)	8	0.07 (0.03, 0.13)	9	0.12 (0.05, 0.23)
Back/side	0	0.00 (0.00, 0.07)	0	0.00 (0.00, 0.03)	3	0.04 (0.01, 0.12)
Chest/ribs/abdomen	0	0.00 (0.00, 0.07)	0	0.00 (0.00, 0.03)	2	0.03 (0.00, 0.10)
Pelvis/hips/groin/upper leg	6	0.11 (0.04, 0.24)	2	0.07 (0.00, 0.06)	5	0.07 (0.02, 0.15)
Knee	2	0.04 (0.00, 0.13)	6	0.05 (0.02, 0.11)	6	0.08 (0.03, 0.17)
Lower leg/ankle/foot	1	0.02 (0.00, 0.10)	3	0.02 (0.01, 0.07)	6	0.08 (0.03, 0.17)
Other	0	0.00 (0.00, 0.07)	0	0.00 (0.00, 0.03)	0	0.00 (0.00, 0.05)
Missing/unknown	3	0.06 (0.01, 0.16)	20	0.17 (0.10, 0.28)	16	0.21 (0.10, 0.45)
Injury type						
Contusion	5	0.09 (0.03, 0.22)	6	0.05 (0.02, 0.11)	5	0.07 (0.02, 0.15)
Concussion	20	0.37 (0.25, 0.56)	33	0.27 (0.20, 0.38)	10	0.13 (0.07, 0.26)
Joint/ligament sprain/dislocation	0	0.00 (0.00, 0.07)	10	0.08 (0.04, 0.18)	14	0.19 (0.10, 0.35)
Fracture	1	0.02 (0.00, 0.10)	5	0.04 (0.01, 0.10)	9	0.12 (0.05, 0.23)
Muscle strain/tendinitis	7	0.13 (0.05, 0.27)	3	0.02 (0.01, 0.07)	8	0.11 (0.05, 0.21)
Abrasion/bleeding/burn/cut/blister	0	0.00 (0.00, 0.07)	1	0.01 (0.00, 0.05)	1	0.01 (0.00, 0.07)
Other	0	0.00 (0.00, 0.07)	1	0.01 (0.00, 0.05)	0	0.00 (0.00, 0.05)
Missing/unknown	2	0.04 (0.00, 0.13)	20	0.17 (0.10, 0.28)	21	0.28 (0.14, 0.54)

^a^ Crude rates per 1000 practice-hours with 95% CIs adjusted for cluster by team. Exact CIs were calculated when number was less than 10.

The results of the multiple multilevel Poisson regression models evaluating each outcome are summarised in Table 5. There was no evidence of non-linearity with weight by itself or within any age group. Female players had a significantly higher rate of injury (incidence rate ratio (IRR) = 1.57; 95% CI: 1.18–2.08) and injury resulting in >7 days of time-loss (IRR = 1.66; 95% CI: 1.19–2.32) than male players (despite 431/599 female players in leagues disallowing body checking). Policy not permitting bodychecking was associated with a 56% lower rate of injury (IRR = 0.44; 95% CI: 0.35–0.55) and a 59% lower rate of injury resulting in >7 days of time-loss (IRR = 0.41; 95% CI: 0.31–0.54). Having a previous injury within the last 12 months and lifetime concussion history were both associated with higher rates of injury and injury with >7 days of time-loss. Relative to forwards, goaltenders had a lower rate of injury (IRR = 0.54; 95% CI: 0.40–0.72) and injury with >7 days of time-loss (IRR = 0.63; 95% CI: 0.45–0.89), while there was no difference in injury rates between forwards and defensive players. Relative to the under-13 age group, under-18 age groups had higher rates of game-related injury (IRR = 1.23; 95% CI: 0.89–1.70) and injury resulting in >7 days of time-loss (IRR = 1.45; 95% CI: 0.99–2.13), but these results were not statistically significant. Year of play, level of play, and player weight were not associated with either game-related outcomes. Female sex (IRR = 1.90; 95% CI: 1.10–3.28) and lifetime concussion history (IRR = 1.55; 95% CI: 1.09–2.20) were the only factors significantly associated with a higher rate of practice-related injury. When comparing the estimates of the models between all injury resulting in >7 days of time-loss with the sensitivity analysis restricting to only musculoskeletal injury with >7 days of time loss (Table S1 in supplemental content), the results were generally consistent. Of note in the musculoskeletal restricted model, under-18 players had significantly higher rates relative to under-13 players, while the effects for females (relative to males) and those with a previous concussion history were no longer statistically significant (but point estimates remained higher for those groups). The point estimate for sub-elite players was below 1 (suggesting lower rates of more severe musculoskeletal injury) but remained non-significant.

**Table 5.** Adjusted incidence rate ratios for injury outcomes in youth ice hockey players.

	Incidence rate ratio (95% CI)		
Potential risk factor	Game-related injury	Game-related injury >7 days of time-loss	Practice-related injury
Age group			
Under-13	1 (reference)	1 (reference)	1 (reference)
Under-15	1.06 (0.78, 1.41)	1.07 (0.76, 1.51)	0.81 (0.46, 1.43)
Under-18	1.23 (0.89, 1.70)	1.45 (0.99, 2.13)	0.90 (0.45, 1.78)
Bodychecking policy			
Permitted	1 (reference)	1 (reference)	1 (reference)
Not permitted	0.44 (0.35, 0.55)^†^	0.41 (0.31, 0.54)^†^	0.84 (0.51, 1.40)
Year of play			
First	1 (reference)	1 (reference)	1 (reference)
Second	1.00 (0.87, 1.15)	1.07 (0.90, 1.27)	1.06 (0.76, 1.49)
Third	1.01 (0.75, 1.36)	1.05 (0.74, 1.49)	1.28 (0.66, 2.48)
Level of play			
Elite (top 20–30%)	1 (reference)	1 (reference)	1 (reference)
Sub-elite (lower 70–80%)	1.07 (0.90, 1.29)	1.15 (0.94, 1.41)	1.19 (0.82, 1.73)
Sex			
Male	1 (reference)	1 (reference)	1 (reference)
Female	1.57 (1.18, 2.08)^†^	1.66 (1.19, 2.32)^†^	1.90 (1.10, 3.28)^†^
Player weight	1.00 (0.99, 1.00)	0.99 (0.98, 1.00)	1.00 (0.98, 1.02)
Previous injury in the last year^a^			
No	1 (reference)	1 (reference)	1 (reference)
Yes	1.46 (1.26, 1.70)^†^	1.44 (1.18, 1.75)^†^	1.26 (0.87, 1.82)
Previous concussion^b^			
No	1 (reference)	1 (reference)	1 (reference)
Yes	1.41 (1.23, 1.62)^†^	1.50 (1.27, 1.78)^†^	1.55 (1.09, 2.20)^†^
Position			
Forward	1 (reference)	1 (reference)	1 (reference)
Defense	1.00 (0.87, 1.14)	1.05 (0.89, 1.25)	0.93 (0.65, 1.34)
Goalie	0.54 (0.40, 0.72)^†^	0.63 (0.45, 0.89)^†^	1.09 (0.65, 1.83)

^a^ The covariate ‘previous injury in the last year’ includes any concussion that occurred in the previous 12 months.

^b^ The covariate ‘previous concussion’ includes any concussion without a date limit.

^†^ Statistically significant at *p* < .05.

## Discussion

This study investigated potential risk factors associated with rates of game and practice-related injury in Canadian youth ice hockey players using data from a large, prospective cohort. Consistent with other studies, our results suggest that one of the strongest factors associated with game-related injury rates is policy permitting bodychecking [7,10–14,27]. Collectively, the substantial evidence demonstrating lower game-related injury rates following policy disallowing bodychecking suggests significant public health impact. Research examining injury risk after policy restricting bodychecking has shown no overall unintended injury consequences related to fewer years of bodychecking experience for adolescent players in leagues permitting bodychecking [9,15,16]. This counters the argument made in the ice hockey community that bodychecking should be implemented earlier to protect players from injury when they are older.

Our results suggest that females had a 57% greater rate of game-related injury, a 66% greater rate of game-related injury resulting in >7 days of time-loss, and a 90% greater rate of practice-related injury relative to male players. A higher proportion of females who participated in ‘female-only’ leagues were injured relative to females playing in leagues on predominately male-based teams. Potentially, differences may exist between the sociocultural context of females playing in male-dominated leagues and females in ‘female-only’ leagues related to injury reporting or differences within gameplay affecting risk of injury [28]. While ‘female-only’ leagues do not permit bodychecking at any age or level of play, these leagues have been noted to have high levels of body contact (i.e. positional play that includes angling players into the boards) which, when combined with the fast speeds of play, may result in inadvertent collisions and opportunities for injury [29]. Future studies are needed to better understand why females are at greater risk of injury to inform prevention programs [28].

Players with a previous injury history in the last 12 months and a lifetime history of concussion had significantly higher rates of game-related injury and injury with >7 days of time-loss. This finding is consistent with several previous youth ice hockey studies as well as research in other sports [7–10,15,16,22,30]. Also of note was that players with a lifetime concussion history had a significant 55% higher rate of practice-related injury. Goalies had a significantly lower rate of both game-related injury outcomes relative to forwards, similar to the results of prior studies [7,8,10,12–14,16]. While under-18 leagues did not have significantly higher rates of injury than under-13 leagues, the trend of higher injury rates in increasingly older age groups is consistent with previous studies [8,10,13,14].

Year of play, level of play, and player weight were not related with any injury outcomes in the current study. First year players in under-13 leagues have been suggested to have a higher risk of injury relative to second year players [8,10], but year of play was not significantly associated in older age groups [13,14]. Studies have varied on whether level of play is associated with injury risk and for which levels [7,9,10,12,15,16,20]. This may be related to different populations between studies and categorisations of level of play. Previous studies suggest that injury rates were highest for players in the lowest weight category [7,12], while other studies suggest an increased risk in heavier players [13]. For the under-15 age group, higher point estimates for all injury and concussion outcomes were seen for lighter and heavier categories relative to the middle category [16], but this relationship was not seen in under-18 players [15]. These conflicting findings may be due to different populations of interest and different categorisations or analysis of weight (i.e. examined continuously or categorically).

The estimates from the model that was restricted to only musculoskeletal injury with >7 days of time loss were largely consistent with the model examining all injury with >7 days of time loss (i.e. including concussion). These findings are also generally consistent with concussion specific outcomes in this cohort; however, sub-elite players were associated with higher rates of concussion outcomes [31], but not injury rates in the current study. Players in the under-18 age group had a significantly higher rate of musculoskeletal injury with >7 days of time loss than under-13 and under-15 (IRR = 1.60; 95% CI: 1.13–2.26, as obtained from linear combinations from the restricted model), but age group was not related to game-related concussion outcomes [31]. Factors related to other specific injury types or locations remain an opportunity for further evaluation.

Limitations of the Safe2Play cohort include self-reporting of covariate data, which may change over the course of the season (e.g. position of play). As previously suggested, several factors may affect the return to play decision after an injury such as importance of the game, motivation, player personality characteristics and parental influence [13,14]. Each of these may affect the precision of the IRR estimates of the injury outcome based on time-loss. Survivor bias is possible in sport-related longitudinal cohorts when players stop their sport participation after an injury leading to loss to follow-up in the cohort. The players that continue to participate may have a lower underlying risk profile than those that stopped playing and this remains an area for future research [9,15]. Selection bias is possible based on teams not willing to participate; however, the main reason for non-participation was the inability to identify someone on the team willing to record playing exposure and injury details. As such, it is unlikely that non-participation would be related to any exposure variable and injury outcomes. The multiple multilevel models included a random effect at a team level to account for clustering effects but did not include a random effect at the individual level. While models accounting for both levels of random effects were attempted, these crossed-effects are known for their prohibitively long computational times when adjusting for several covariates [32].

## Conclusions

Based on data from a large 5-year longitudinal cohort in Canadian youth ice hockey, several factors were associated with game-related injury and injury resulting in >7 days of time-loss. The factors associated with higher injury rates included being female, playing in older age groups, having a history of injury within the previous 12 months, and a lifetime history of concussion. Factors associated with a lower rate of injury included the goaltender position and playing in leagues where policy disallowed bodychecking. Bodychecking policy continues to be the most reported modifiable risk factor in youth ice hockey and policy disallowing bodychecking can have the greatest public health impact in reducing the burden of injury. Future research in ice hockey should target females and female-specific injury prevention strategies.

## Supplementary Material

Supplemental Material

## Data Availability

All data relevant to the study are included in the article or uploaded as supplemental information.
