# Supplementary material for: Safe2Play in youth ice hockey: injury profile and risk factors in a 5-year Canadian longitudinal cohort study
Source: Ann Med. 2024 Aug 27;56(1):2385024. doi: 10.1080/07853890.2024.2385024 (PMC11351362; doi:10.1080/07853890.2024.2385024)
Supplement: Supplemental Material [file IANN_A_2385024_SM4689.docx]

Supplemental Content.

Table S1 Adjusted incidence rate ratios for game-related musculoskeletal injury with >7 days of time loss in youth ice hockey players

|  | Incidence Rate Ratio (95% CI) |
| --- | --- |
| Potential Risk Factor | **Game-related musculoskeletal injury >7 days of time-loss** |
| Age Group  Under-13  Under-15  Under-18 | 1 (Reference)  1.87 (0.90, 3.58)  2.99 (1.38, 6.44)**†** |
| Bodychecking Policy  Permitted  Not permitted | 1 (Reference)  0.25 (0.15, 0.40)**†** |
| Year of play  First  Second  Third | 1 (Reference)  1.02 (0.78, 1.33)  1.02 (0.63, 1.67) |
| Level of play  Elite (top 20-30%)  Sub-elite (lower 70-80%) | 1 (Reference)  0.80 (0.59, 1.09) |
| Sex  Male  Female | 1 (Reference)  1.44 (0.78, 2.69) |
| Player Weight | 0.99 (0.98, 1.01) |
| Previous injury in the last year^a^  No  Yes | 1 (Reference)  1.45 (1.08, 1.95)**†** |
| Previous concussion^b^  No  Yes | 1 (Reference)  1.24 (0.96, 1.62) |
| Position  Forward  Defense  Goalie | 1 (Reference)  0.91 (0.69, 1.19)  0.53 (0.30, 0.95)**†** |

^a^The covariate “previous injury in the last year” includes any concussion that occurred in the previous 12 months.

^b^The covariate “previous concussion” includes any concussion without a date limit.

† Statistically significant at p<0.05.
